# Congenital Talipes Equino-Varus

**Published:** 1907-09-14

**Authors:** 


### CONGENITAL TALIPES EQUINO-VARUS.

### III. TREATMENT.

(*Continued from page* 579.)

No mention has been made of the use of wrenches in the treatment of talipes, because it is the writer's opinion that it is quite possible to perform with manual force alone everything that can be done with a wrench, without running the risks that necessarily accompany the use of these formidable engines. The commonest complication that is liable to occur after their employment is the appearance of a pressure sore at the spot where the lever has been applied to the skin. It is true that this can be avoided by having the instrument carefully padded; but besides this, the skin of the foot may give way or the bones of the leg be fractured, or, worse still, an epiphysis may be separated. This last accident is very troublesome; to secure accurate union in cases of separated epiphyses, so that no deformity results, is difficult enough in a normal limb, but in a limb already distorted from its natural shape it is well-nigh impossible.

### Non-Operative Treatment.

The rigid or resistant form of talipes whose anatomical features have already been described is rarely seen in any but the poorer class of patients. Among the well-to-do the deformity is generally treated in infancy. It occurs in children who have been allowed to walk on the foot without any attempt having been made to correct the deformity, and is very commonly seen in the out-patient department of a general hospital. Even in these cases much improvement may be obtained by a long course of gradual treatment, extending over as long a time as twelve or eighteen months. The line of treatment is much the same as in simpler cases, except that it should commence by a month's complete rest, during which time the pain due to muscular spasm disappears and any sore places on the foot heal up. Then manipulation, tenotomies, and retentive apparatus should be given a thorough trial. It is only fair to add that although much improvement often takes place, complete restitution of the foot is hardly ever obtained on account of the permanent osseous deformity which has developed.

### Operative Treatment.

For this reason, and on account of the tedious nature of this form of treatment in advanced cases, it is generally advisable to adopt some more drastic measure, such as open operation. Many kinds of tarsotomy and tarsectomy have been described and advocated. That which is best known by name, although in the writer's experience it is the one least commonly practised, is Phelps' operation. An incision is made from a point half-way between the apex of the internal malleolus and the tuberosity of the scaphoid downwards and outwards to the junction of the inner two-thirds with the outer third of the sole. It is carried deeply, so as to divide the plantar fascia, the abductor hallucis, the tibialis posticus and anticus, the internal lateral ligament and the calcaneo-scaphoid ligament. The foot is then manipulated into the normal position and fixed there with plaster-of-Paris, and a gaping cavity is left which is allowed to granulate up. The operation is rarely successful in getting rid of the deformity permanently, because the fibrous tissue which replaces the granulations tends to go on contracting until eventually the original state of affairs is reproduced with the additional disfigurement of a deep furrow in the sole. To obviate this some surgeons graft skin over the granulating area, while others recommend the insertion of a wedge-shaped plate of decalcified bone into the gap at the time of the operation. Further than this the operation deals only with the soft parts, and does not make any attempt to modify the altered condition of the bones of the tarsus; it is therefore an incomplete procedure in long-standing cases of talipes.

### Cuneiform Tarsectomy.

The second alternative, the operation of cuneiform tarsectomy, is the exact converse of Phelps' operation. An incision is made along the outer border of the foot from the middle of the os calcis to that of the fifth metatarsal, and a second one, about one and a half inches long, at right angles to the first and directed towards the sole. The soft parts are raised from the bones with a periosteum elevator, and a wedge-shaped piece of the tarsus removed with a saw or chisel, irrespective of the articulations. Its size should be so calculated that the foot is in position when the gap is closed. The result is not satisfactory, and relapse is common. The most satisfactory operation in advanced cases of talipes is removal of the astragalus. It is best done by means of a median vertical incision over the prominent bone. The tendons are retracted to each side. The ankle and astragalo-scaphoid joints are opened, and the astragalus itself seized with lion-forceps and drawn from side to side as the remaining ligamentous structures are divided. Some surgeons advocate a transverse incision across the front of the ankle, and division of the tendons, which are united after the removal of the bone. This has nothing to commend it. The vertical incision gives the surgeon quite sufficient access to the parts, because the nature of the deformity renders the astragalus prominent. This is undoubtedly the most satisfactory of the operations at present in vogue. The deformity is corrected and cannot recur, and there is no necessity for the subsequent wearing of mechanical contrivances. But it must not be understood that astragalectomy is advocated as a routine measure. Every case must be judged on its own merits. If the varus element is more advanced than the equinus, better results can be obtained by a cuneiform tarsectomy; but in the great majority of cases the main deformity is due to the prominence of the astragalus on the dorsum of the foot, which prevents flexion at the ankle-joint, and it is in these cases that astragalectomy is especially indicated.

